# A case report of a young woman with pulmonary emboli and a right coronary artery-to-coronary sinus fistula

**DOI:** 10.1093/ehjcr/ytae130

**Published:** 2024-03-15

**Authors:** V Karthik, Chinedum Anosike, Christos Zivlas

**Affiliations:** Warrington Hospital, Warrington and Halton Hospitals NHS Foundation Trust, Lovely Lane, Warrington WA5 1QG, UK; Warrington Hospital, Warrington and Halton Hospitals NHS Foundation Trust, Lovely Lane, Warrington WA5 1QG, UK; Warrington Hospital, Warrington and Halton Hospitals NHS Foundation Trust, Lovely Lane, Warrington WA5 1QG, UK

**Keywords:** Cardiac magnetic resonance imaging, Case report, Coronary artery fistula, CT coronary angiography, Pulmonary embolism, Right coronary artery, Transthoracic echocardiogram

## Abstract

**Background:**

Coronary artery fistulae are rare cardiovascular anomalies that can present with atypical symptomatology and therefore pose diagnostic challenges, especially in young patients.

**Case summary:**

A 34-year-old woman presented with left-sided pleuritic chest pain, haemoptysis, and flu-like symptoms. Initial evaluation revealed multiple left-sided pulmonary emboli, and her transthoracic echocardiography showed turbulent flow in a dilated coronary sinus. A right coronary artery (RCA) to coronary sinus fistula was confirmed by computed tomography coronary angiogram. The patient was treated with lifelong anticoagulation, and a subsequent stress cardiac magnetic resonance imaging did not show inducible myocardial ischaemia. As such, the patient was managed conservatively.

**Discussion:**

Utilization of multi-modality imaging is of utmost importance for diagnostic and therapeutic purposes in coronary artery fistulae. In this case report, our patient presented with unprovoked pulmonary emboli, which could be caused by the turbulent flow and stasis, due to the RCA-to-coronary sinus fistula.

**Learning points**

- Understand the importance of echocardiography in excluding the existence of an unroofed coronary sinus.
- Recognize the diagnostic tools, such as computerized tomography coronary angiography and cardiac magnetic resonance imaging, used in the evaluation of coronary artery fistulae.
- Consider the potential relationship between coronary artery fistulae and pulmonary embolism.

## Introduction

A coronary artery fistula, whether arising congenitally or acquired through life, is an abnormal connection between a coronary artery and a cardiac chamber (known as a coronary-cameral fistula) or vessel (known as a coronary arteriovenous fistula). Most commonly, a fistula terminates in the right ventricle, right atrium, or pulmonary artery.^1^ This occurs rarely, in approximately 0.1–0.2% of the population.^2,3^ Most of these patients become symptomatic in the fourth to sixth decades of life and commonly present with exertional dyspnoea and fatigue.^4^ Often, a small fistula does not have important haemodynamic consequences for the patient.^5^ In this report, we present the case of a 34-year-old woman presenting to the hospital with several weeks of fever and a cough. She was found to have multiple left-sided pulmonary emboli and a right coronary artery (RCA) to coronary sinus fistula as confirmed by computed tomography coronary angiogram (CTCA).

## Summary figure

**Figure:**
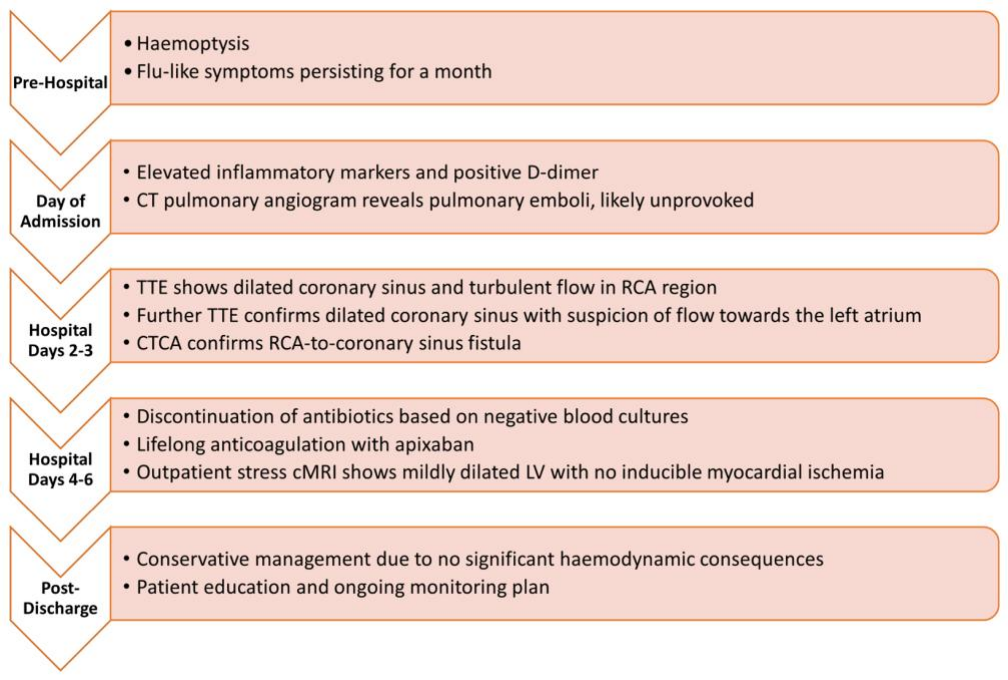


## Case presentation

A 34-year-old Caucasian woman presented to the emergency department at our institution, with left-sided pleuritic chest pain and haemoptysis for 5 days on the background of being unwell for a month with flu-like symptoms that included fatigue, myalgia, and night sweats. She denied any recent foreign travel or any intravenous drug use in the past. On admission, her blood pressure was 173/100 mmHg (this subsequently reduced to 105/62 mmHg during admission), and her heart rate was 91 b.p.m. She was saturating 100% on room oxygen and was apyrexial at 36.2°C. On lung auscultation, there were mild inspiratory crepitations heard along the left base of the lung. On cardiac auscultation, a systolic murmur was heard over the aortic area that was louder on expiration. On further clinical examination, there were no enlarged neck or axillary lymph nodes. She tested negative for COVID-19 and her initial electrocardiogram showed normal sinus rhythm with good R-wave progression, narrow QRS, normal QRS axis, normal PR and QTc intervals, and no ischaemic changes. Her chest X-ray revealed patchy airspace opacification at the left base of the lung that was thought to be infective in nature.

The patient had no known medical history but a family history of a sister who underwent EP ablation for atrioventricular nodal re-entrant tachycardia and suffered a deep vein thrombosis after pregnancy.

**Figure 1:**
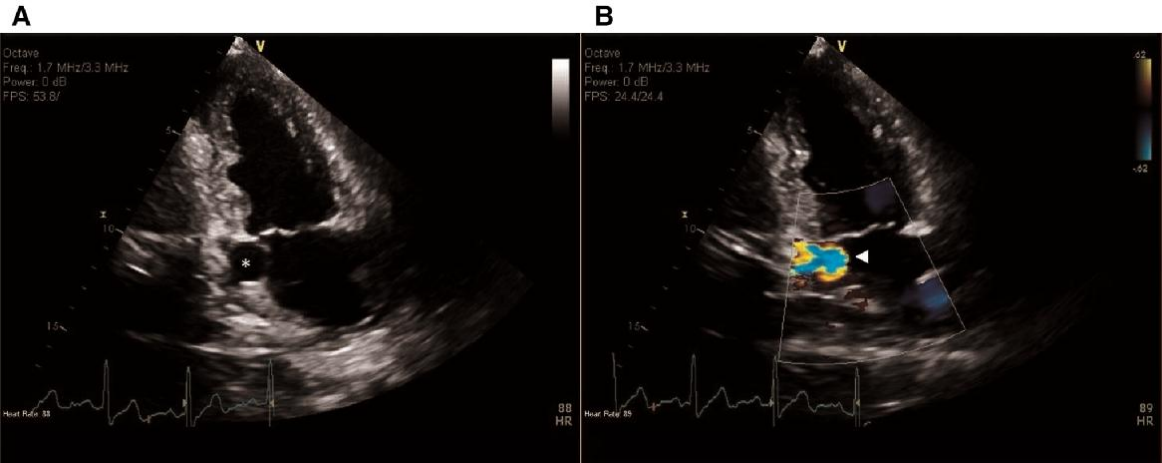
Initial transthoracic echocardiography. (*A*) Two-dimensional apical two-chamber view, showing a dilated coronary sinus (asterisk). (*B*) Colour Doppler imaging showed aliasing of colour in the coronary sinus, indicative of high velocity flow (arrowhead).

**Figure 2:**
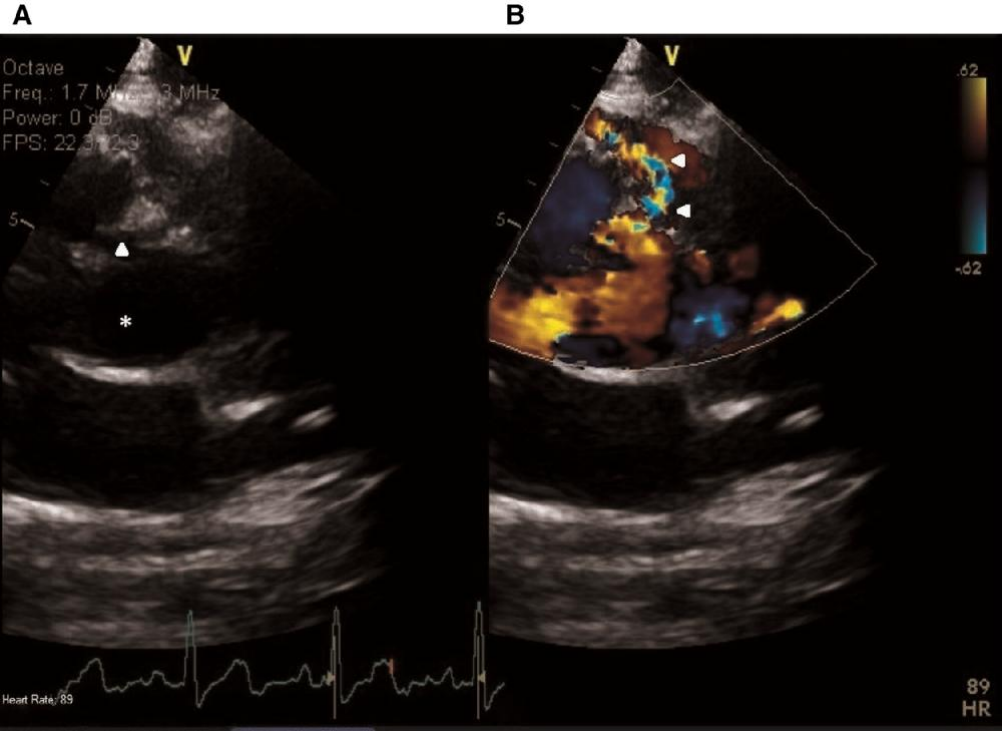
Initial transthoracic echocardiography. Two-dimensional parasternal long-axis, simultaneous colour Doppler view. (*A*) The aortic root (asterisk) and the prominent origin of the right coronary artery (arrowhead) are depicted. (*B*) Colour Doppler imaging showed turbulent flow, probably down to the right coronary artery (arrowheads).

**Figure 3:**
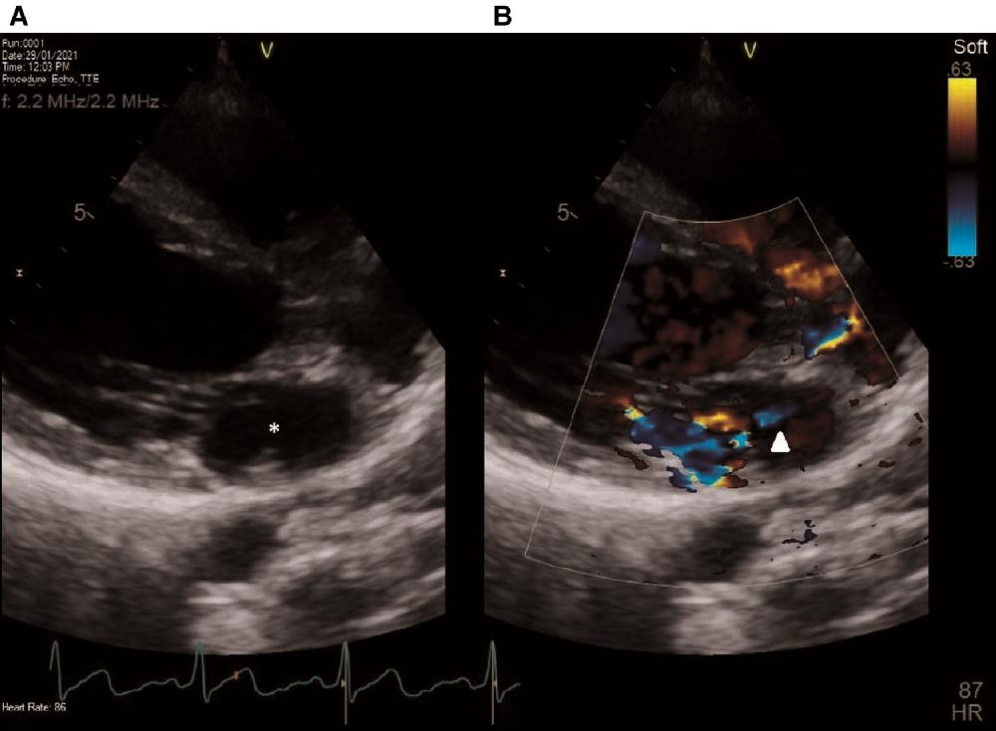
Subsequent transthoracic echocardiography. Two-dimensional parasternal modified long-axis, simultaneous colour Doppler view. (*A*) The left atrium was depicted (asterisk). (*B*) There appeared to be a jet directed inside the left atrium (arrowhead), raising the suspicion of an unroofed coronary sinus.

**Figure 4:**
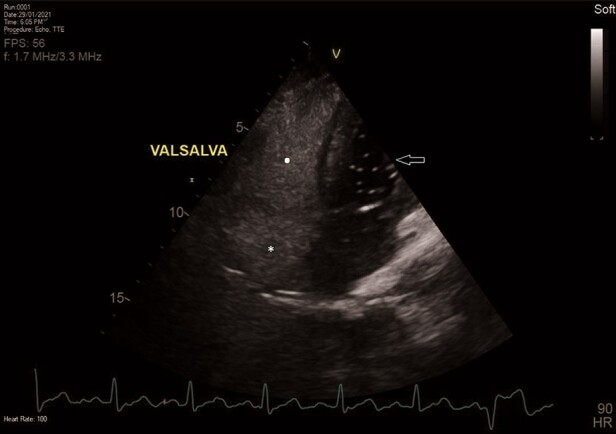
Subsequent transthoracic echocardiography. Two-dimensional apical two-chamber view, after administration of agitated normal saline and Valsalva manoeuvre. The right atrium (asterisk) and the right ventricle (white dot) are fully opacified. However, only a small number of bubbles appeared in the left atrium and left ventricle (arrow). This was indicative of a patent foramen ovale (PFO).

**Figure 5:**
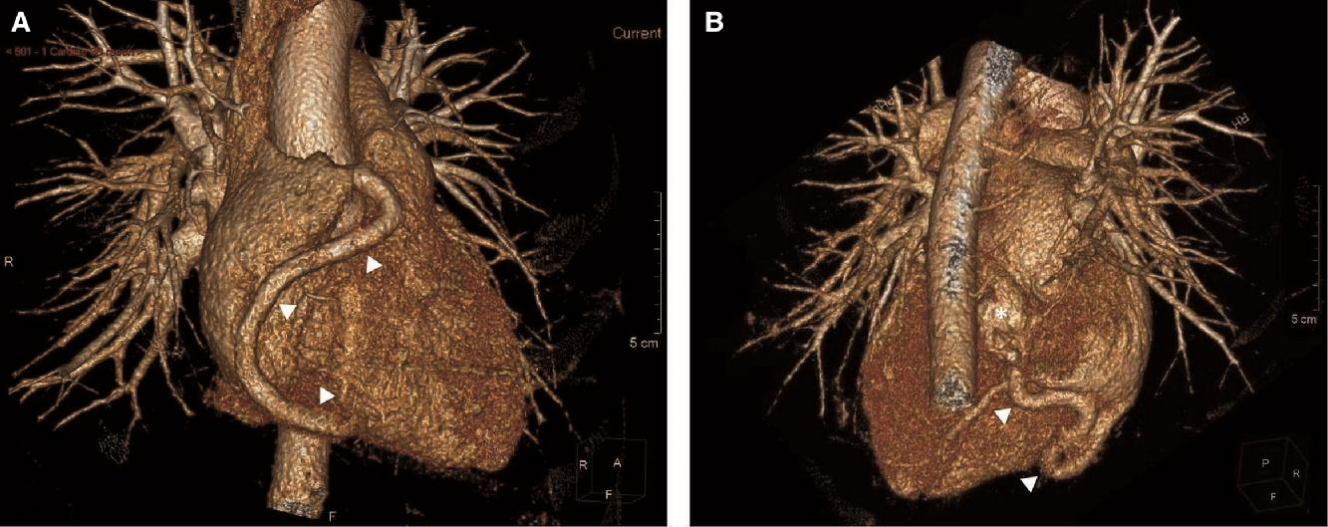
Three-dimensional image reconstruction of the computed tomography coronary angiogram. (*A*) Right anterolateral view showing the course of the right coronary artery, which was dilated (arrowheads). (*B*) The dominant right coronary artery (arrowheads) was tortuous and followed a posterior–inferior course, where it finally abutted the coronary sinus (asterisk).

Initial blood tests revealed raised inflammatory markers, with an initial C-reactive protein of 71 mg/L (normal <5 mg/L) and erythrocyte sedimentation rate of 43 mm/h (normal range 1–9 mm/h, when adjusted for patient demographics), with C4 complement of 0.44 g/L (normal range 0.10–0.40 g/L) and a positive D-dimer test (qualitatively reported, with normal range 0–500 µg/L). The patient’s white blood cell count was 9.6 × 10^9^/L on admission and peaked at 11 × 10^9^/L (normal range 3.8–11.0 × 10^9^/L) 4 days later, before dropping back to normal levels, and the urine sample provided returned negative for bacterial growth while revealing normal white and red blood cell counts. Computed tomography pulmonary angiogram revealed probable sub-acute pulmonary emboli that had mostly resolved but with several peripheral pulmonary infarcts. There were no pathological mediastinal, hilar, or axillary lymph nodes identified, no malignancy of the chest and the upper abdominal viscera and no destructive bone lesion. Blood cultures returned no growth. Initial transthoracic echocardiography (TTE) revealed no obvious vegetations but showed a dilated coronary sinus with aliasing on colour Doppler imaging (*Figure 1*; Supplementary material online, *Videos S1* and *S2*) and turbulent flow in the region of the RCA (*Figure 2*; Supplementary material online, *Video S3*). A further TTE was done the following day and revealed good left ventricular and right ventricular systolic function with non-dilated ventricles, no evidence of infective endocarditis, but confirmed a dilated coronary sinus with suspicion of flow towards the left atrium (*Figure 3*; Supplementary material online, *Video S4*). Due to suspicion of an unroofed coronary sinus, a bubble transthoracic echocardiogram was performed with administration of agitated saline through a left antecubital fossa vein. However, this was not confirmed, and no significant right-to-left shunt was detected. Only a small number of bubbles were noted in the left atrium after a Valsalva manoeuvre, indicating a patent foramen ovale (*Figure 4*; Supplementary material online, *Video S5*). A CTCA was performed, revealing an RCA-to-coronary sinus fistula (*Figure 5*).

The patient was started on intravenous antibiotics upon admission with initial suspicion of infective endocarditis. However, this was discontinued after negative blood cultures and the absence of obvious vegetations identified on TTE. With the confirmation of multiple pulmonary emboli, the patient was then started on lifelong, therapeutic anticoagulation with apixaban and referred for thrombophilia screening, which was ultimately negative. A transabdominal and transvaginal ultrasound of her pelvis performed a few days after discharge excluded any obvious gynaecological malignancy.

A stress cardiac magnetic resonance imaging scan with adenosine was performed in an outpatient setting after discharge. This did not reveal any stress-inducible myocardial ischemia. The left ventricle was mildly dilated (end-diastolic volume index 103 mL/m^2^—normal values 62–96 mL/m^2^) but with normal ejection fraction (left ventricular ejection fraction - LVEF 59%). The right ventricle was not dilated and had good systolic function. The pulmonary artery was not dilated.

With no significant haemodynamic consequences or complications of the RCA-to-coronary sinus fistula, the patient was managed conservatively.

## Discussion

We describe the case of a young female patient who was diagnosed with multiple left-sided pulmonary emboli and an incidental finding of RCA-to-coronary sinus fistula.

There are several complications associated with coronary artery fistulae that relate to their size. Small fistulae tend to have a good prognosis without any interventions, whereas medium-to-large fistulae can be complicated by angina or myocardial infarction, arrhythmias, heart failure, or endocarditis.^5^ The preferred diagnostic modality is CTCA and has been found to be more accurate than conventional angiography.^6^ Indications for percutaneous or surgical closure include being symptomatic, complications as aforementioned and a significant shunt. In a case of a 47-year-old woman with fistulae originating from both the right coronary and left circumflex coronary arteries and terminating in the coronary sinus, percutaneous closure was attempted before surgical closure was pursued, owing to her presentation with congestive heart failure.^6^

In the literature, there appear to be two cases with septic pulmonary emboli resulting from infective endocarditis as a complication of coronary artery fistulae.^7,8^ However, the direct causative relationship between coronary artery fistulae and pulmonary emboli has not been thoroughly investigated. In our patient, an association between the RCA-to-coronary sinus fistula and pulmonary emboli, due to the turbulent flow and stasis, cannot be excluded.

In conclusion, this case report highlights the diagnostic and management challenges posed by a rare presentation of an RCA-to-coronary sinus fistula in a young female patient with multiple pulmonary emboli. While the fistula had no significant haemodynamic consequences, its association with pulmonary emboli should be considered. Congenital coronary anomalies and fistulae should always be excluded in young patients with atypical symptoms and an inexplicable presentation, such as unprovoked pulmonary emboli.

## Supplementary Material

ytae130_Supplementary_Data

## Data Availability

The authors confirm that the data supporting the findings of this case report are available within the manuscript and its Supplementary material online. Further details can be requested by contacting the corresponding authors.

## Lead author biography

Dr V. Karthik holds an MBBS degree from Barts and the London School of Medicine and Dentistry. Having completed foundation training with Warrington & Halton Hospitals NHS Trust, he aspires to specialize in cardiology, with a keen interest in pursuing interventional cardiology or electrophysiology.

## Supplementary material

**Consent:** The authors confirm that written consent has been obtained from the patient for the submission and publication of this case report, including images and associated test, in accordance with the Committee on Publication Ethics (COPE) guidance.

**Funding:** None declared.
